# To Know or Not to Know? Theta and Delta Reflect Complementary Information about an Advanced Cue before Feedback in Decision-Making

**DOI:** 10.3389/fpsyg.2016.01556

**Published:** 2016-10-06

**Authors:** Jing Wang, Zhaofeng Chen, Xiaozhe Peng, Tiantian Yang, Peng Li, Fengyu Cong, Hong Li

**Affiliations:** ^1^Brain Function and Psychological Science Research Center, Shenzhen UniversityShenzhen, China; ^2^School of Psychology, South China Normal UniversityGuangzhou, China; ^3^Department of Biomedical Engineering, Faculty of Electronic Information and Electrical Engineering, Dalian University of TechnologyDalian, China

**Keywords:** delta, theta, reinforcement learning, agent, time–frequency.

## Abstract

To investigate brain activity during the reinforcement learning process in social contexts is a topic of increasing research interest. Previous studies have mainly focused on using electroencephalograms (EEGs) for feedback evaluation in reinforcement learning tasks by measuring event-related potentials. Few studies have investigated the time–frequency (TF) profiles of a cue that manifested whether a following feedback is available or not after decision-making. Moreover, it remains unclear whether the TF profiles of the cue interact with different agents to whom the feedback related. In this study we used the TF approach to test EEG oscillations of the cue stimuli in three agents (‘Self’, ‘Other’, and ‘Computer’) conditions separately. The results showed that the increased central-posterior delta power was elicited by the feedback unavailable cues more so than with the feedback available cue within 200–350 ms after the onset of the cue, but only in the self-condition. Moreover, a frontal-central theta oscillation had enhanced power when following the feedback unavailable cue as opposed to the feedback available cue across three agencies. These findings demonstrated that the cue for knowing an outcome produced reward prediction error-like signals, which were mirrored by the delta and theta oscillations during decision-making. More importantly, the present study demonstrated that the theta and delta oscillations reflected separable components of the advanced cue processing before the feedback in decision-making.

## Introduction

It is important for human beings to learn from external feedback after making a decision for maximizing reward. In the past two decades, many researchers have adopted varied decision-making tasks, in which feedback was available to facilitate participant behaviors (for reviews, see Walsh and Anderson, 2012; Ullsperger et al., 2014). Nevertheless, it is quite common that a person cannot get access to the feedback of his/her own decision-making in reality. Therefore, it would be interesting to investigate how the brain response to a cue that informs whether the decision-makers’ feedback will be shown or not. Despite plenty of literatures discussing the neural bases of feedback learning, however, few studies have focused on the brain activity changes in human subjects when they receive a cue which indicated feedback information will either be available, or not.

Given that the feedback information plays a key role in a trial-and-error learning task, participant curiosity about feedback will be evoked when feedback information is unavailable, according to the information-gap theory (Loewenstein, 1994). Loewenstein (1994) proposed an information-gap theory that epistemic curiosity is aroused when an individual realizes a difference between “what one wants to know” and “what one knows”. Kang et al. (2009) have found that epistemic curiosity activates reward circuitry in a functional magnetic resonance imaging (fMRI) study. One of our previous studies shows that the participants preferred to know others’ results in a gambling task, even when to know this information was costly (Han et al., 2012). Thus, it is plausible that curiosity will not be satisfied when feedback information is unavailable. According to reinforcement learning theories, the reward prediction error (RPE), *i.e*. differences between expected and obtained reinforcements, can be used to adjust associations between actions and corresponding reward in decision-making (Schultz, 1997; Holroyd and Coles, 2002). In a broader sense, RPE could be elicited in many situations in which a mismatch between expected and actual outcomes occurs (Kuss et al., 2011) and predict the “goodness” of on-going events (Holroyd and Coles, 2002). Taking these two lines of research together, we hypothesized that the cue of missing feedback will generate RPE during reinforcement learning task and this signal can be detected by electroencephalograph (EEG) activities in the brain.

By recording EEG activity on the scalps of human participants, previous event-related brain potentials (ERP) and time–frequency (TF) studies have linked the feedback-related negativity (FRN) component, frontal midline theta, and delta oscillations with reward predication error signals originating from the mid-brain dopamine system (Bernat et al., 2011, 2015; Foti et al., 2014; Li et al., 2016; Pornpattananangkul and Nusslock, 2016). The FRN was observed in the frontal-central region in a 200–350 ms time window after feedback stimulus presented and showed larger amplitude following negative feedback than following positive feedback (Miltner et al., 1997; Holroyd and Coles, 2002). Due to the component overlapping issue in traditional ERP studies, the FRN has been considered to be affected by the P300 component (or later positive component) which arises right after the former FRN (Sambrook and Goslin, 2015).

In our previous paper, we exploited a gambling task with three agencies (Self, Other, and PC) involving, and manipulating, a cue which indicated whether the results of three agencies’ gambling were unavailable or not (Han et al., 2013). We observed that larger later positivity component (LPC) was associated with unavailable cues compared to available cues. Moreover, the LPC in the Other condition was correlated with the interpersonal curiosity trait in participants (Han et al., 2013). In fact, we hypothesized that the FRN component could be the component of interest in that study (Han et al., 2013, p. 46). One possibility behind the vanished FRN component might be the overlapping from other late component as mentioned above.

The TF method which focused on spectral characteristics should help to separate the FRN from P300 (Bernat et al., 2011). Earlier studies revealed that the P300 is composed mainly of activity in the delta (<3 Hz) band (Bernat et al., 2007; Gilmore et al., 2010) while the FRN is composed largely of activity in the theta (4–8 Hz) range (Cohen et al., 2011). In addition to these issues in FRN studies in particular, traditional ERP approaches also lack the ability to detect the rich, complex information, about oscillatory activity that varies in phase from trial-to-trial (Cohen et al., 2007, 2011). Hence, we used the TF method to explore the multi-dimensional neural dynamics of feedback information cue processing. Recent studies have linked the theta and delta frequencies with RPE in decision-making tasks (Foti et al., 2014; Bernat et al., 2015). Therefore, we mainly focused on theta and delta power in the present study.

Studies focused on feedback-guided learning have consistently found that increasing theta power (4–8 Hz) was associated with feedback that was worse than expected, i.e., negative RPE (Cohen et al., 2007; Marco-Pallares et al., 2008; Hajihosseini et al., 2012). Although these finding have often been replicated, there were also inconsistent findings around whether the theta power was sensitive to RPE in particular or unexpected events in general (Cohen et al., 2007; Doñamayor et al., 2012; Hajihosseini and Holroyd, 2013). With a dynamic reward-learning task and associated computational model, Cavanagh et al. (2011) found that medial-frontal theta was correlated with unsigned prediction error but has an asymmetrical sensitivity to negative events. Interestingly, the same group has found that the medial and lateral frontal theta corresponded to the degree of negative RPE and positive RPE in the service of behavioral adjustment (Cavanagh et al., 2010). Taken these together, medial frontal theta oscillation seems to be a good candidate index for the processing of negative RPE in our study, in which, the two types of cue occurred with equal probability.

The converged evidence showed that both waking and sleep delta waves mainly originate from the medial frontal cortical regions (for a review, see Knyazev, 2012), however, delta activity was shown to be concentrated in more posterior regions on the scalp (for a review, see Güntekin and Başar, 2015). Delta oscillations have been implicated in the motivational relevance of the task and the salience of the target stimulus (Knyazev, 2007, 2012) and appear to be associated with reward processing (Knyazev, 2007; Cavanagh, 2015). In a dynamic reinforcement learning task, Cavanagh found that delta activity at different times reflected RPE and state prediction error separately (Cavanagh, 2015). More specifically, early delta activity, which constitutes reward positivity, may correspond to a surprising reward signal, while later delta activity, which contributed to the P300 component, appeared to associate with behavioral adjustments. The finding that delta frequency was sensitive to positive RPEs was also reported in a recent study, which used principal components analysis (Sambrook and Goslin, 2016).

Based on the aforementioned literature, we reanalyzed the data from our previous paper (Han et al., 2013) using the TF approach and focused on the oscillation profiles of cues which indicated whether the feedback will be available or not. We also compared the TF distributions in three agents’ conditions in order to show whether the cue effect is only self-relevant or related to any agent in general. We hypothesized that the cue for showing feedback will elicit positive RPE, while that for no feedback will generate negative RPE. Moreover, these RPEs could be reflected by the medial frontal theta and delta oscillation. In addition, a recent study, in which the researcher applied the TF method to a classical gambling task, has demonstrated that the theta and delta frequencies reflected different functional significances in the outcome evaluation (Bernat et al., 2015). According to this finding, we also hypothesized that the theta and delta measures may have different sensitivities to agent and cue-type.

## Materials and Methods

Previous non-overlapping results from this dataset are reported elsewhere (Han et al., 2013). For more detailed information about the experimental design, see Han et al. (2013).

### Participants

Nineteen subjects participated in the experiment as volunteers. One participant’s data was excluded because of excessive movement artifacts. Thus, the data from 18 participants (eight males) with ages ranging from 20 to 25 (*M* = 22.1 and *SD* = 1.4) were taken for TF analysis. All participants were healthy and right-handed. They all had normal, or corrected-to-normal, vision and gave informed written consent before participation. This study was approved by the local Ethics Committee.

### Experimental Procedure

The participants were informed that they would participate in a three-agent (Self, another participant called “Other”, and a computer, called “PC”) on-line gambling game. Unknown to the real participants, the “Other” participant was pretended by a research assistant and his/her behavior was simulated by computer. The “Other” participant was a stranger with the same gender to the real participant and they were introduced to each other before the experiment. Each trial began with a 500 ms white fixation cross against a black background, followed by a picture of two golden eggs which was displayed for 800 ms on the screen. Then a phrase appeared on the two eggs to indicate which agent’s turn it was next. “Your turn” means it was the participants’ turn to make a decision between these two eggs, while “A’s turn” and “PC’s turn” represented the other person’s turn and the PC’s turn to make a selection, correspondingly. Three agents could press either “F” or “J” to select the left or right egg. Thereafter, a 500 ms confirmatory cue appeared with a red circle to confirm the selection, followed by a blank screen, which lasted randomly from 600 to 1000 ms. Then, a yellow circle without a cross (available cue), or with a cross inside (unavailable cue), was presented on the screen to indicate whether the participant would see the feedback from this trial or not. Participants were told that whether feedback was given or not did not affect their cumulative monetary gain. For trials with an unavailable cue, a “?” mark would appear instead of the outcome.

There were 360 critical trials in total. The experiment was conducted with two (available, unavailable) by three (Self, Other, and PC) within-subject design and each condition was repeated 60 times. The order of the three agents’ actions was randomized at the trial level. The participants had a 1-min break after each group of 72 trials. All stimuli were presented by E-Prime Version 1.1 software on a computer.

### Data Acquisition

Brain electrical activity was recorded at 64 scalp sites using tin electrodes mounted in an elastic cap (Brain Product, Munich, Germany), with a ground electrode placed on the frontal midline and references placed on the left and right mastoids. Vertical electrooculograms (EOGs) were recorded supra-orbitally and infra-orbitally relative to the left eye. The horizontal EOG was recorded as the difference in activity from the right vs. the left orbital rim. The impedances of all of the electrodes were less than 10 kΩ. The EEG and EOG were amplified using a 0.05–100 Hz band pass and continuously digitized at 500 Hz/channel for off-line analysis. Note that the following ERP and TF analysis were time-locked to the onset of the cue stimuli before the final feedback.

### Data Analysis

Electroencephalograph data were imported and processed using EEGLAB (Delorme and Makeig, 2004). Continuous EEG data were band-pass filtered at between 1 and 40 Hz. EEG epochs were extracted using a window analysis time of 2000 ms (1000 ms pre-stimulus and 1000 ms post-stimulus) and baseline corrected using the pre-stimulus time interval. Trials contaminated by eye-blinks and movements were corrected using an independent component analysis (ICA) algorithm (Delorme and Makeig, 2004). In all datasets, individual eye movements, showing a large EOG channel contribution and a frontal scalp distribution, were clearly observed in the removed independent components. After pre-processing, these data were submitted to further TF analysis.

We were interested in the identification and characterization of oscillatory activities induced by each stimulus. A wavelet transform was used for the TF analysis in this study. The EEG data from each single trial were convoluted by complex Morlet wavelets *W*(*t,f*_0_) (Kronland-Martinet et al., 1987) having a Gaussian shape both in the time domain *SD*σ_t_ and in the frequency domain *SD*σ_f_ around its central frequency $f_{0}\,\;:\;W(t,f_{0})=A\cdot \exp(-t^{2}/2\sigma_{t}^{2})\cdot \exp(2i\pi f_{0}t),$ with σ_f_ = 1/2σ_t_. A wavelet family is characterized by a constant ratio (*f*_0_/σ_f_), which should be chosen in practice to be greater than five (Grossmann et al., 1989). The wavelet family used here was defined by *f*_0_/σ_f_ = 7, with *f*_0_ ranging from 1 to 30 Hz. The time resolution of this method, therefore, increases with frequency, whereas the frequency resolution decreases. After that, the TF representations (absolute value of the wavelet transform) of single-trial EEG data were averaged over single-trials given each channel, each subject, and each stimulus. Subsequently, the data from -200 to 800 ms were taken for further analysis to avoid the edge effect of in the wavelet transform used here. The baseline was then corrected for each frequency bin.

For the statistical analysis, the FRN-like amplitude, the theta power within 200–350 ms and 400–700 ms was measured at FCz where these activities peak (Holroyd and Coles, 2002; Li et al., 2010, 2015, 2016), whereas the delta power within 200–350 ms was measured at Cz and the delta power within 400–700 ms was calculated at Pz based on the present scalp distributions of the delta activity.

## Results

### ERP Results

Note that we have reported the ERP results in a previous study (Han et al., 2013); however, we used a narrow frequency band (0.01–16 Hz) to filter the original data. To facilitate the appropriate TF analysis using a wavelet transform in this study, the continuous EEG data were filtered by the 1–40 Hz bandpass filter during pre-processing.

**FIGURE 1:**
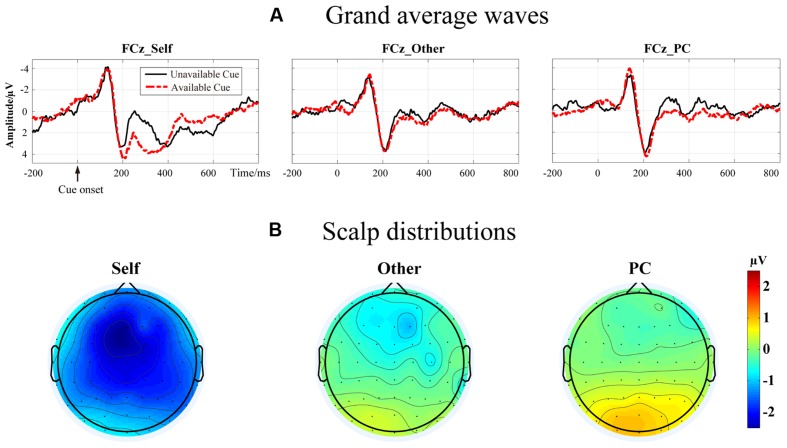
**(A)** The grand average waves elicited by a unavailable cue (red) and an available cue (green) in Self, Other, and PC conditions at FCz. **(B)** The corresponding scalp distribution of three difference waves between the event-related potential elicited by unavailable cue and available cue in three conditions separately.

As showed in **Figure 1**, the ERP data were measured as the mean value of the difference wave between available and unavailable conditions within the 200–350 ms time window at FCz. These data were submitted to one way ANOVA with agent (Self, Other, and PC) as within-subject variables. The results showed that the main effect of agent was significant, *F*(2,34) = 14.91, *p* < 0.001, and η^2^ = 0.47. The pair-wise comparison suggested that the difference wave in the Self condition (-2.58 ± 0.46 μV) was significantly larger than that in the Other condition (-0.75 ± 0.5 μV and *p* < 0.001), and PC condition (-0.26 ± 0.3 μV and *p* < 0.001). However, there was no significant difference between the difference wave in the Other condition and PC condition (*p* = 0.2).

### TF Results within the 200–350 ms Time Window

**FIGURE 2:**
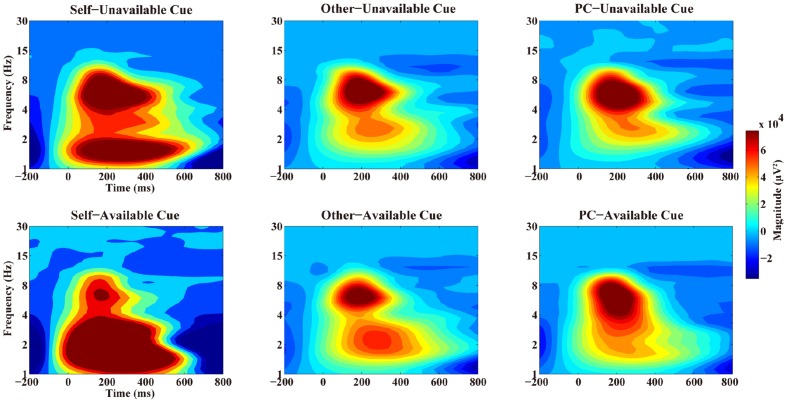
**Time–frequency representation of two types of cues in three agents’ conditions at FCz**. The upper panel showed the time–frequency representation of Unavailable cue in three agents’ condition while the lower panel showed the time–frequency representation of Available cue in three conditions separately.

**FIGURE 3:**
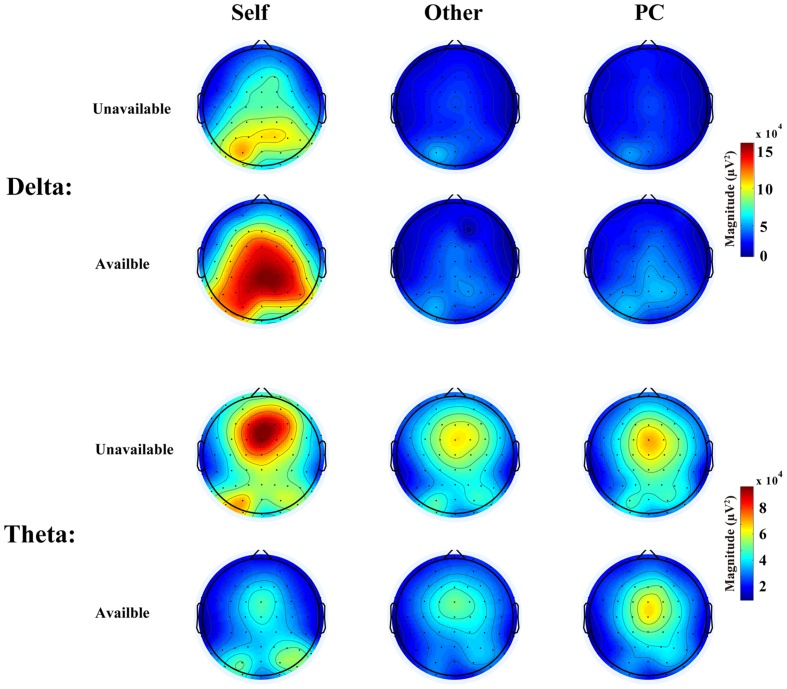
**Scalp distributions of delta and theta elicited by the two types of cues in three agents’ conditions in the 200–350 ms time window**.

The TF representations and corresponding scalp distributions are shown in **Figures 2** and **3**. A three-way ANOVA analysis was carried out on the theta power at FCz with agent (Self, Other, and PC), and cue-type (unavailable and available) as within-subject variables. The results showed that the main effect of cue-type was significant, *F*(1, 17) = 11.24, *p* < 0.005, and η^2^ = 0.40. Pair-wise comparisons revealed that the theta power in the unavailable cue condition (*M* = 7.74 × 10^4^ and *SEM* = 1.14 × 10^4^) was significantly stronger than that in the available cue condition (*M* = 5.42 × 10^4^, *SEM* = 7.6 × 10^3^, and *p* < 0.005).The main effect of agent was not significant, *F*(1.6,28.5) = 1.36, *p* = 0.27, and η^2^ = 0.07. The interaction effect between cue-type and agent did not reach a significant level, *F*(2,34) = 2.66, *p* = 0.09, and η^2^ = 0.14. The main statistical results were shown in **Table 1**.

**Table 1:** Statistical results: theta and delta power in two time-windows.

	200–350 ms		400–700 ms	
	Theta	Delta	Theta	Delta
Agent	*F* = 1.36	*F* = 11.03^∗∗^	*F* = 3.4	*F* = 8.73^∗∗^
Cue-type	*F* = 11.24^∗∗^	*F* = 4.96^∗^	*F* = 11.45^∗∗^	*F* < 1
Agent × Cue-type	*F* = 2.66	*F* = 6.04^∗^	*F* = 3.09	*F* = 2.15

*^∗^*p* < 0.05 and ^∗∗^*p* < 0.01*.

**FIGURE 4:**
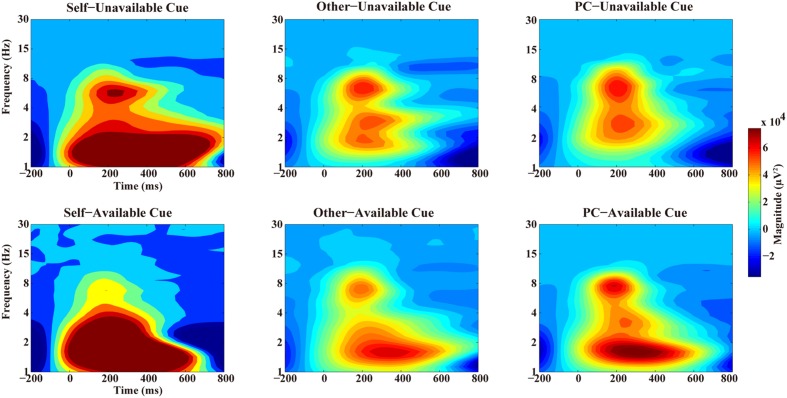
**Time–frequency representation of two types of cues in three agents’ conditions at Pz**. The **Upper panel** showed the time–frequency representation of Unavailable cue in three agents’ condition while the **Lower panel** showed the time–frequency representation of Available cue in three conditions separately.

**FIGURE 5:**
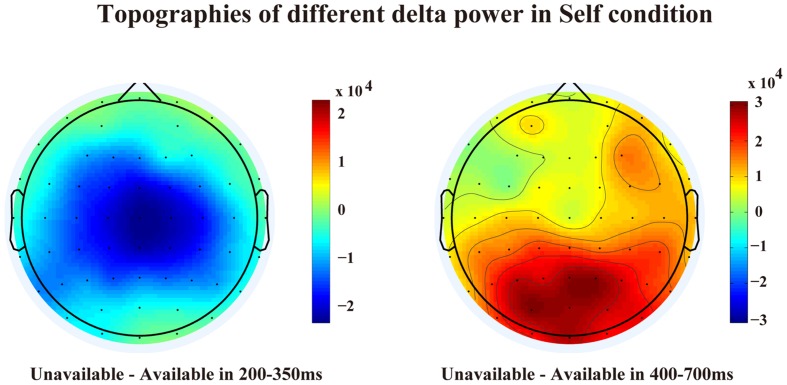
**Scalp distributions of different delta power between unavailable cue and available cue in the Self condition**.

The TF representations and corresponding scalp distributions are shown in **Figures 4** and **5**. A three-way ANOVA analysis was also conducted on the delta power at Cz with agent (Self, Other, and PC) and cue-type (unavailable and available) as independent variables. As shown in **Figure 3**, the main effect of agent reached a significant level, *F*(1.2,20) = 11.03, *p* = 0.002, and η^2^ = 0.39. The following test suggested that the delta power in the Self-condition (*M* = 1.13 × 10^5^ and *SEM* = 2.33 × 10^4^) was significantly larger than that in the Other condition (*M* = 3.98 × 10^4^, *SEM* = 7.24 × 10^3^, and *p* < 0.003), and PC condition (*M* = 4.27 × 10^4^, *SEM* = 9.78 × 10^3^, and *p* < 0.005). However, the delta power was not significantly different between the Other, and the PC, conditions (*p* = 0.7). The main effect of cue-type also reached a significant level, *F*(1,17) = 4.96, *p* < 0.05, and η^2^ = 0.23. Pair-wise comparison showed that the available cue induced a larger delta power (*M* = 8.06 × 10^4^ and *SEM* = 1.48 × 10^4^) than the unavailable cue (*M* = 4.99 × 10^4^ and *SEM* = 1.13 × 10^4^), *p* < 0.05. More importantly, the interaction effect between agent and cue-type was significant, *F*(1.4,23.2) = 6.04, *p* < 0.02, and η^2^ = 0.26. The following analysis suggested that the delta power following an available cue (*M* = 1.6 × 10^5^ and *SEM* = 3.38 × 10^4^) was significantly larger than for an unavailable cue (*M* = 9.23 × 10^4^, *SEM* = 2.5 × 10^4^, and *p* < 0.02) only in the Self-condition, however, there was no notable difference between the delta power elicited by an available cue and an unavailable cue in the Other condition (*p* = 0.46), and PC condition (*p* = 0.45).

### TF Results within the 400–700 ms Time Window

Based on the ERP results in Han et al. (2013) and the ERP waves as shown in **Figure 1**, we have also analyzed the theta and delta profiles within the 400–700 ms time window. As mentioned above, a three-way ANOVA was carried out on the theta power at FCz with agent (Self vs. Other vs. PC) and cue-type (unavailable vs. available) as independent variables. Results revealed that the main effect of agent was not significant, *F*(1.4,24.2) = 3.4, *p* = 0.07, and η^2^ = 0.17. However, the main effect of cue-type reached a significant level, *F*(1,17) = 11.45, *p* = 0.004, and η^2^ = 0.4. Pair-wise comparison showed that a larger theta power was observed following an unavailable cue (*M* = 1.05 × 10^4^ and *SEM* = 3.65 × 10^3^) than after an available cue (*M* = 1.21 × 10^3^ and *SEM* = 2.37 × 10^3^). There was no other significant interaction effect found (*p* = ns).

The statistical results for delta at Pz showed that the main effect of agent was significant, *F*(1.3,22.7) = 8.73, *p* = 0.004, and η^2^ = 0.34. Pair-wise comparison revealed that the delta power in the Self-condition (*M* = 6.35 × 10^4^ and *SEM* = 1.97 × 10^4^) was notably larger than that in the Other condition (*M* = 1.53 × 10^4^ and *SEM* = 9.9 × 10^3^, *p* = 0.003), and PC condition (*M* = 1.37 × 10^4^, *SEM* = 6.58 × 10^3^, and *p* = 0.01), while there was no significant difference between the latter two conditions (*p* = 0.85). However, the main effect of cue-type was not significant, *F*(1,17) < 1, *p* = 0.9, and η^2^ = 0.001. The interaction effect did not reach a significant level, *F*(1.2,20.4) = 2.15, *p* = 0.16, and η^2^ = 0.11.

## Discussion

In the present work, we have reanalyzed the data from our previous ERP study using a TF approach. Basically, the present work has extended our findings from the ERP results of a cue which manifested whether the outcome of a decision will be presented or not. In the previous study, we found that the LPC amplitude was notably different between the “unavailable” and the “available” conditions and linearly reduced from “Self”, to “Other”, to “PC” conditions (Han et al., 2013). Here, we showed that the main difference of ERP between the “unavailable” and the “available” conditions appeared from about 200 ms following cue presentation. The feature of these difference waves, and their scalp distributions, suggested that the “unavailable” cue elicited an FRN-like effect after 1 Hz high-pass filtering. These results confirmed the current concerns that the FRN effect might have been superimposed by the LPC component which is mainly driven by the power of low-frequency activities (Sambrook and Goslin, 2016). Moreover, the decomposition of EEG activity by TF method allowed us to understand better how the experimental manipulation affected the brain activities when processing these advanced cues.

The present findings firstly expanded current understanding of reinforcement learning processes in such a trial and error task. A large number of studies have focused on the outcome evaluation by time-domain and frequency domain (for reviews, see Ullsperger et al., 2014). Much evidence converges to show that FRN component, delta activity, and medial frontal theta power, reflected the RPE signals in reinforcement learning (Cohen et al., 2011; Cavanagh, 2015; Sambrook and Goslin, 2016). Our design and data reanalysis, to our knowledge, is the first to have shown that the advanced cue before outcome elicited similar RPE signals, which was reflected on frontal central theta frequency and central posterior delta activity within the traditional FRN time window. In fact, Bromberg-Martin and Hikosaka (2009) found that macaque monkeys preferred to seek advance information about the size of a water reward. More importantly, their results showed that the same mid-brain dopamine neurons response to primitive, and the desire for advance, information about upcoming rewards, which they termed as ‘cognitive reward’, was present. In line with this study, another functional MRI study also found that cognitive feedback activated similar dopaminergic regions as monetary reward in an information-integration category learning task (Daniel and Pollmann, 2010). Therefore, we speculated that an advanced cue that indicated the information availability of upcoming monetary reward may have also been processed as a cognitive reward in human learning.

The theta oscillation profiles elicited by the advanced cue replicated current TF studies which focused on negative and positive feedback evaluation in a reinforcement learning task (Marco-Pallares et al., 2008; Cavanagh et al., 2010; Foti et al., 2014; Mas-Herrero and Marco-Pallarés, 2014; Bernat et al., 2015). The enhanced theta band power has been associated with negative RPE compared to positive RPE in reinforcement learning tasks (Marco-Pallares et al., 2008; Cavanagh et al., 2010). Notably, the theta power was divergent between the unavailable cue condition and available cue condition within both of the two time windows analyzed, however, the theta frequency was not sensitive to the agent in the present context. This finding suggested that the theta activity was related to the processing of missing “cognitive reward” information in general. In our design, three agencies have conducted the same gambling task in the laboratory. Therefore, our participants could learn from their own feedback directly, and from Other’s and PC’s feedbacks, by observational learning (Bandura et al., 1966). Additionally, Holroyd and Coles (2002) suggested that the RPE was conveyed from the mid-brain dopamine system to ACC and is used as a “teaching signal” to other regions for the purpose of improving performance (Sambrook and Goslin, 2015). Based on this background, we proposed that the theta oscillation might be associated with an RPE signal which was used to improve learning, including self-relevant and observational learning. Another possibility is that the theta oscillation is sensitive to general negative events, such as the blocking of curiosity and satisfaction for Self, Other’s, and the PC’s results here, and social rejection, in a previous study (Cristofori et al., 2013).

In contrast to the theta frequency, the delta activities revealed different patterns in the 200–350 ms and 400–700 ms time windows. Larger delta power was observed following available cue than after unavailable cue mainly at central electrodes, however, this effect was mainly observed in the Self-condition and only in the 200–350 ms period, which is the classical FRN time window. The central delta activity in the first phase replicated previous findings which demonstrated that enhanced delta power was observed in positive feedback condition than in negative feedback condition (Bernat et al., 2015; Cavanagh, 2015; Li et al., 2016). These results might support the statement that the early central delta oscillation is related to positive RPE signal, which generated from the dopamine system (Cavanagh, 2015).

Interestingly, the delta frequency in the late time window (400–700 ms) reached maximum at posterior sites and only affected by the agent factor. These results firstly demonstrated that the late posterior delta oscillation reflected a different process compared to the early delta. The delta activities in the 400–700 ms could mainly contribute the LPC amplitude which was reported in our previous paper (Han et al., 2013). According to an influential theory, late large positive deflection, such as P3 reflected the activity of the neuromodulatory locus coeruleus–norepinephrine system, which enhance the response to motivationally significant events (Nieuwenhuis et al., 2005; Nieuwenhuis, 2011). Based on this theory, we inferred that the late posterior delta activity also reflected motivational significance of self-relevant information and corresponding attentional involvement. In specific, although the probabilities of cue stimuli are equal among three agents, self-relevant cues are more salient compared to Other’s cue and PC’s cue in such a context. The higher motivational significance of self-relevant information may be driven by ‘engaged curiosity’ as suggested by Panksepp (1998).

Note that Bernat et al. (2015) recently suggested that theta reflects most salient primary feedback while delta is sensitive to both primary and second feedback attributes. Although both their data, and our data, suggested that theta and delta reflect different separable components in reinforcement learning tasks, our study demonstrated that the delta, rather than theta, reflected more salient and self-relevant stimuli. On the other hand, theta power showed sensitivity to all of the negative events across agencies over two time windows (200–350 ms and 400–700 ms). These inconsistent findings may have been due to the different paradigms and phases of interest in the two studies. Additionally, several recent TF studies have found that frontal theta was related to the unsigned RPE rather than negative RPE (Hajihosseini and Holroyd, 2013). Given that the probabilities of the two types of cues were equal in the present study, we could not provide evidence to support this argument, leaving an open question for future study: whether the theta power observed here would be modulated by probability of cognitive reward, or not.

### Limitations and Future Directions

Bromberg-Martin and Hikosaka (2009) suggested that modern reinforcement learning theory should take ‘cognitive reward’ into consideration. Our study further provided electrophysiological evidence from human subjects that ‘cognitive reward’ can generate similar RPE signals, which were indexed here by theta and early delta power, as the primary and second rewards. The processing of the cue information may alarm the system into preparing for future feedback information with the aim of optimizing reinforcement learning. As proposed by Holroyd and Yeung (2012), humans need to learn context-specific sequences of behavior to achieve a final goal through hierarchical reinforcement learning. When a cue was established between decision-making and feedback, the brain may have to adjust the connection between response and reward during reinforcement learning. The processing of an advanced cue may be part of hierarchical reinforcement learning in a special context. The lack of trials undertaken in the final feedback phase in the present study, meant that this hypothesis could not be tested directly by comparing the processing of cue and feedback. Future studies are, therefore, required to test this hypothesis.

## Author Contributions

PL and JW designed the study, JW and XP collected the data, FC, TY, and ZC analyzed the data, and PL and HL wrote the paper.

## Conflict of Interest Statement

The authors declare that the research was conducted in the absence of any commercial or financial relationships that could be construed as a potential conflict of interest. The reviewer BE and handling Editor declared their shared affiliation, and the handling Editor states that the process nevertheless met the standards of a fair and objective review.

## References

[B1] Bandura A. Grusec J. E. Menlove F. L. (1966). Observational learning as a function of symbolization and incentive set. *Child Dev.* 37 499–506. 10.2307/1126674 4165810

[B2] Bernat E. M. Malone S. M. Williams W. J. Patrick C. J. Iacono W. G. (2007). Decomposing delta, theta, and alpha time–frequency ERP activity from a visual oddball task using PCA. *Int. J. Psychophysiol.* 64 62–74. 10.1016/j.ijpsycho.2006.07.015 17027110 PMC2276568

[B3] Bernat E. M. Nelson L. D. Baskin-Sommers A. R. (2015). Time-frequency theta and delta measures index separable components of feedback processing in a gambling task. *Psychophysiology* 52 626–637. 10.1111/psyp.12390 25581491 PMC4398588

[B4] Bernat E. M. Nelson L. D. Steele V. R. Gehring W. J. Patrick C. J. (2011). Externalizing psychopathology and gain–loss feedback in a simulated gambling task: dissociable components of brain response revealed by time-frequency analysis. *J. Abnorm. Psychol.* 120 352–364. 10.1037/a0022124 21319875 PMC3092030

[B5] Bromberg-Martin E. S. Hikosaka O. (2009). Midbrain dopamine neurons signal preference for advance information about upcoming rewards. *Neuron* 63 119–126. 10.1016/j.neuron.2009.06.009 19607797 PMC2723053

[B6] Cavanagh J. F. (2015). Cortical delta activity reflects reward prediction error and related behavioral adjustments, but at different times. *Neuroimage* 110c, 205–216. 10.1016/j.neuroimage.2015.02.007 25676913

[B7] Cavanagh J. F. Figueroa C. M. Cohen M. X. Frank M. J. (2011). Frontal theta reflects uncertainty and unexpectedness during exploration and exploitation. *Cereb. Cortex* 22 2575–2586. 10.1093/cercor/bhr332 22120491 PMC4296208

[B8] Cavanagh J. F. Frank M. J. Klein T. J. Allen J. J. (2010). Frontal theta links prediction errors to behavioral adaptation in reinforcement learning. *Neuroimage* 49 3198–3209. 10.1016/j.neuroimage.2009.11.080 19969093 PMC2818688

[B9] Cohen M. X. Elger C. E. Ranganath C. (2007). Reward expectation modulates feedback-related negativity and eeg spectra. *Neuroimage* 35 968–978. 10.1016/j.neuroimage.2006.11.056 17257860 PMC1868547

[B10] Cohen M. X. Wilmes K. A. Vijver I. V. D. (2011). Cortical electrophysiological network dynamics of feedback learning. *Trends Cogn. Sci.* 15 558–566. 10.1016/j.tics.2011.10.004 22078930

[B11] Cristofori I. Moretti L. Harquel S. Posada A. Deiana G. Isnard J. (2013). Theta signal as the neural signature of social exclusion. *Cereb. Cortex* 23 2437–2447. 10.1093/cercor/bhs236 22875860

[B12] Daniel R. Pollmann S. (2010). Comparing the neural basis of monetary reward and cognitive feedback during information-integration category learning. *J. Neurosci.* 30 47–55. 10.1523/JNEUROSCI.2205-09.2010 20053886 PMC6632509

[B13] Delorme A. Makeig S. (2004). Eeglab: an open source toolbox for analysis of single-trial eeg dynamics including independent component analysis. *J. Neurosci. Methods* 134 21 10.1016/j.jneumeth.2003.10.009 15102499

[B14] Doñamayor N. Schoenfeld M. A. Münte T. F. (2012). Magneto- and electroencephalographic manifestations of reward anticipation and delivery. *Neuroimage* 62 17–29. 10.1016/j.neuroimage.2012.04.038 22561022

[B15] Foti D. Weinberg A. Bernat E. M. Proudfit G. H. (2014). Anterior cingulate activity to monetary loss and basal ganglia activity to monetary gain uniquely contribute to the feedback negativity. *Clin. Neurophysiol.* 126 1338–1347. 10.1016/j.clinph.2014.08.025 25454338 PMC4385748

[B16] Gilmore C. S. Malone S. M. Bernat E. M. Iacono W. G. (2010). Relationship between the P3 event-related potential, its associated time-frequency components, and externalizing psychopathology. *Psychophysiology* 47 123–132. 10.1111/j.1469-8986.2009.00876.x 19674392 PMC2860032

[B17] Grossmann A. Kronland-Martinet R. Morlet J. (1989). “Reading and understanding continuous wavelets transforms,” in *Wavelets Time Frequency Methods and Phase Space*, eds Combes J. M. Grossmann A. Tchamitchian P. (New York: Springer), 2–20.

[B18] Güntekin B. Başar E. (2015). Review of evoked and event-related delta responses in the human brain. *Int. J. Psychophysiol.* 103 43–52. 10.1016/j.ijpsycho.2015.02.001 25660301

[B19] Hajihosseini A. Holroyd C. B. (2013). Frontal midline theta and N200 amplitude reflect complementary information about expectancy and outcome evaluation. *Psychophysiology* 50 550–562. 10.1111/psyp.12040 23521513

[B20] Hajihosseini A. Rodríguez-Fornells A. Marco-Pallarés J. (2012). The role of beta-gamma oscillations in unexpected rewards processing. *Neuroimage* 60 1678–1685. 10.1016/j.neuroimage.2012.01.125 22330314

[B21] Han C. Li P. Feng T. Y. Li H. (2012). People’s current circumstances modulate interpersonal curiosity. *J. Psychol. Sci.* 35 1435–1439.

[B22] Han C. Li P. Warren C. Feng T. Litman J. Li H. (2013). Electrophysiological evidence for the importance of interpersonal curiosity. *Brain Res.* 1500 45–54. 10.1016/j.brainres.2012.12.046 23333374

[B23] Holroyd C. B. Coles M. G. H. (2002). The neural basis of human error processing: reinforcement learning, dopamine, and the error-related negativity. *Psychol. Rev.* 109 679–709. 10.1037/0033-295X.109.4.679 12374324

[B24] Holroyd C. B. Yeung N. (2012). Motivation of extended behaviors by anterior cingulate cortex. *Trends Cogn. Sci.* 16 122–128. 10.1016/j.tics.2011.12.008 22226543

[B25] Kang M. J. Hsu M. Krajbich I. M. Loewenstein G. McClure S. M. Wang J. T. (2009). The wick in the candle of learning: epistemic curiosity activates reward circuitry and enhances memory. *Psychol. Sci.* 20 963–973. 10.1111/j.1467-9280.2009.02402.x 19619181

[B26] Knyazev G. G. (2007). Motivation, emotion, and their inhibitory control mirrored in brain oscillations. *Neurosci. Biobehav. Rev.* 31 377–395. 10.1016/j.neubiorev.2006.10.004 17145079

[B27] Knyazev G. G. (2012). EEG delta oscillations as a correlate of basic homeostatic and motivational processes. *Neurosci. Biobehav. Rev.* 36 677–695. 10.1016/j.neubiorev.2011.10.002 22020231

[B28] Kronland-Martinet R. Morlet J. Grossmann A. (1987). Analysis of sound patterns through wavelet transforms. *Intern. J. Pattern Recognit. Artif. Intell.* 1 273–302. 10.1142/S0218001487000205

[B29] Kuss K. Falk A. Trautner P. Elger C. E. Weber B. Fliessbach K. (2011). A reward prediction error for charitable donations reveals outcome orientation of donators. *Soc. Cogn. Affect. Neurosci.* 8 216–223. 10.1093/scan/nsr088 22198972 PMC3575724

[B30] Li P. Baker T. E. Warren C. Li H. (2016). Oscillatory profiles of positive, negative and neutral feedback stimuli during adaptive decision making. *Int. J. Psychophysiol.* 107 37–43. 10.1016/j.ijpsycho.2016.06.018 27378537

[B31] Li P. Jia S. Feng T. Liu Q. Suo T. Li H. (2010). The influence of the diffusion of responsibility effect on outcome evaluations: electrophysiological evidence from an ERP study. *Neuroimage* 52 1727–1733. 10.1016/j.neuroimage.2010.04.275 20452440

[B32] Li P. Song X. Wang J. Zhou X. Li J. Lin F. (2015). Reduced sensitivity to neutral feedback versus negative feedback in subjects with mild depression: evidence from event-related potentials study. *Brain Cogn.* 100 15–20. 10.1016/j.bandc.2015.08.004 26432379

[B33] Loewenstein G. (1994). The psychology of curiosity: a review and reinterpretation. *Psychol. Bull.* 116 75–98. 10.1037/0033-2909.116.1.75

[B34] Marco-Pallares J. Cucurell D. Cunillera T. García R. Andrés-Pueyo A. Münte T. F. (2008). Human oscillatory activity associated to reward processing in a gambling task. *Neuropsychologia* 46 241–248. 10.1016/j.neuropsychologia.2007.07.016 17804025

[B35] Mas-Herrero E. Marco-Pallarés J. (2014). Frontal theta oscillatory activity is a common mechanism for the computation of unexpected outcomes and learning rate. *J. Cogn. Neurosci.* 26 447–458. 10.1162/jocn_a_00516 24188368

[B36] Miltner W. H. Braun C. H. Coles M. G. (1997). Event-related brain potentials following incorrect feedback in a time-estimation task: evidence for a “generic” neural system for error detection. *J. Cogn. Neurosci.* 9 788–798. 10.1162/jocn.1997.9.6.788 23964600

[B37] Nieuwenhuis S. (2011). “Learning, the P3, and the locus coeruleus-norepinephrine system,” in *Neural Basis of Motivational and Cognitive Control*, eds Mars R. Sallet J. Rushworth M. Yeung N. (Oxford: Oxford University Press), 209–222.

[B38] Nieuwenhuis S. Aston-Jones G. Cohen J. D. (2005). Decision making, the p3, and the locus coeruleus-norepinephrine system. *Psychol. Bull.* 131 510–532. 10.1037/0033-2909.131.4.510 16060800

[B39] Panksepp J. (1998). *Affective Neuroscience.* New York: Oxford University Press.

[B40] Pornpattananangkul N. Nusslock R. (2016). Willing to wait: elevated reward-processing eeg activity associated with a greater preference for larger-but-delayed rewards. *Neuropsychologia* 91 141–162. 10.1016/j.neuropsychologia.2016.07.037 27477630 PMC5110616

[B41] Sambrook T. D. Goslin J. (2015). A neural reward prediction error revealed by a meta-analysis of ERPs using great grand averages. *Psychol. Sci.* 141 213–235. 10.1037/bul0000006 25495239

[B42] Sambrook T. D. Goslin J. (2016). Principal components analysis of reward prediction errors in a reinforcement learning task. *Neuroimage* 124 276–286. 10.1016/j.neuroimage.2015.07.032 26196667

[B43] Schultz W. (1997). Dopamine neurons and their role in reward mechanisms. *Curr. Opin. Neurobiol.* 7 191–197. 10.1016/S0959-4388(97)80007-4 9142754

[B44] Ullsperger M. Fischer A. G. Nigbur R. Endrass T. (2014). Neural mechanisms and temporal dynamics of performance monitoring. *Trends Cogn. Sci.* 18 259–267. 10.1016/j.tics.2014.02.009 24656460

[B45] Walsh M. M. Anderson J. R. (2012). Learning from experience: event-related potential correlates of reward processing, neural adaptation, and behavioral choice. *Neurosci. Biobehav. Rev.* 36 1870–1884. 10.1016/j.neubiorev.2012.05.008 22683741 PMC3432149

